# Increase in EPI vaccines coverage after implementation of intermittent preventive treatment of malaria in infant with Sulfadoxine -pyrimethamine in the district of Kolokani, Mali: Results from a cluster randomized control trial

**DOI:** 10.1186/1471-2458-11-573

**Published:** 2011-07-18

**Authors:** Alassane Dicko, Sidy O Toure, Mariam Traore, Issaka Sagara, Ousmane B Toure, Mahamadou S Sissoko, Alpha T Diallo, Christophe Rogier, Roger Salomon, Alexandra de Sousa, Ogobara K Doumbo

**Affiliations:** 1 Malaria Research and Training Center, Department of Epidemiology of Parasitic Diseases, Faculty of Medicine Pharmacy and Dentistry, University of Bamako, P.O. Box 1805 Bamako, Mali; 2 Department of Public Health, Faculty of Medicine Pharmacy and Dentistry, University of Bamako, P.O. Box 1805 Bamako, Mali; 3 UNICEF Mali, BP: 96, Bamako, Mali; 4 Institut de Recherche Biomédicale des Armées IRBA - ex-IMTSSA & UMR6236-URMITE, Allée du Médecin colonel Jamot, Parc du Pharo, BP60109, 13262 Marseille cedex 07, France; 5 Institut de Santé Publique, d'Épidémiologie et de Développement, Université Victor Segalen Bordeaux 2, Case 11 146 Rue Léo Saignat, 33076 Bordeaux Cedex - France; 6 Special Programme for Research and Training in Tropical Diseases, World Health Organization, 20 Avenue Appia, 121 Geneva 27, Switzerland

**Keywords:** malaria, intermittent preventive treatment, sulphadoxine-pyrimethamine, expanded program of immunization

## Abstract

**Background:**

Even though the efficacy of Intermittent Preventive Treatment in infants (IPTi) with Sulfadoxine-Pyrimethamine (SP) against clinical disease and the absence of its interaction with routine vaccines of the Expanded Immunization Programme (EPI) have been established, there are still some concerns regarding the addition of IPTi, which may increase the work burden and disrupt the routine EPI services especially in Africa where the target immunization coverage remains to be met. However IPTi may also increase the adherence of the community to EPI services and improve EPI coverage, once the benefice of strategy is perceived.

**Methods:**

To assess the impact of IPTi implementation on the coverage of EPI vaccines, 22 health areas of the district of Kolokani were randomized at a 1:1 ratio to either receive IPTi-SP or to serve as a control. The EPI vaccines coverage was assessed using cross-sectional surveys at baseline in November 2006 and after one year of IPTi pilot-implementation in December 2007.

**Results:**

At baseline, the proportion of children of 9-23 months who were completely vaccinated (defined as children who received BGG, 3 doses of DTP/Polio, measles and yellow fever vaccines) was 36.7% (95% CI 25.3% -48.0%). After one year of implementation of IPTi-SP using routine health services, the proportion of children completely vaccinated rose to 53.8% in the non intervention zone and 69.5% in the IPTi intervention zone (P <0.001).

The proportion of children in the target age groups who received IPTi with each of the 3 vaccinations DTP2, DTP3 and Measles, were 89.2% (95% CI 85.9%-92.0%), 91.0% (95% CI 87.6% -93.7%) and 77.4% (95% CI 70.7%-83.2%) respectively. The corresponding figures in non intervention zone were 2.3% (95% CI 0.9% -4.7%), 2.6% (95% CI 1.0% -5.6%) and 1.7% (95% CI 0.4% - 4.9%).

**Conclusion:**

This study shows that high coverage of the IPTi can be obtained when the strategy is implemented using routine health services and implementation results in a significant increase in coverage of EPI vaccines in the district of Kolokani, Mali.

**Trial Registration:**

ClinicalTrials.gov NCT00766662

## Background

Malaria is one of biggest killer of infants and children in sub-Saharan Africa. In the absence of a vaccine against malaria, the intermittent preventive treatment of malaria in infant (IPTi) has been developed to reduce the burden of malaria in infants. IPTi consists on the administration of a curative dose of antimalarials at the time of EPI vaccination during the first year of life regardless of the presence of symptoms or infection [1]. Several randomized control studies of IPTi with Sulfadoxine-pyrimethamine (SP) in different parts of Africa have shown that the strategy is efficacious in preventing clinical episodes of malaria by 30% and incidence of anemia by 20% [2] although no efficacy was found in an area where the resistance to SP was 82% [3]. The strategy is effective and well accepted by the communities [4-7]. It has also been established that the administration of SP at the time of EPI vaccines shows no negative interaction with these vaccines [8-10] and no safety issue was found [11,12]. A consortium was established in 2003 to generate scientific evidence required to inform and speed up the process of going from strategy into policy [1]. In 2009 the WHO has recommended IPTi-SP as policy for malaria control in areas where the malaria transmission is moderate- to -high and where the parasite resistance to SP is not high (prevalence of dhps 540 mutation <50%) [13]. Even though the efficacy, the absence of interaction with EPI vaccines and safety of IPTi are well established, there are still concerns that addition of IPTi will result in an increase in the work burden and will disrupt the routines EPI services especially in Africa where the target EPI vaccines coverage remains to be met. However IPTi may also increase the adherence of the community to EPI services and improve EPI coverage, once the benefit of the strategy is perceived [14,15].

Mali has one of the highest infant mortality rate in the world estimated at 119 per 1000 (http://www.unicef.org/french/infobycountry/mali_statistics.html) along with a high malaria burden. Malaria is reported by the health centers as a primary cause of the morbidity and mortality according routine health reports [16] and is supported by more rigorous cohort studies [17,18]. P. falciparum resistance to SP remains low in Mali [19,20] and the prevalence of mutation dhps 540 is less than 5% [21,22]. The proportion of children completely vaccinated with EPI vaccines in Mali remained below 50% according to health surveys in 2001 and 2006 [23,24]. This study aimed to evaluate the impact of the IPTi-SP implementation on the coverage of EPI vaccines after one year of intervention in a context of a large pilot implementation of health services in the district of Kolokani, Mali.

## Methods

### Study site

The study was conducted in the district of Kolokani, Mali. The district of Kolokani is an administrative subdivision of the region of Koulikoro, in Mali. The town of Kolokani is located at about 140 km north of Bamako. The district covers 14,380 km^2^, divided into 22 health sub-districts. Each health sub-district is composed of several villages. The total population was 208,317 inhabitants with children under 1 year representing about 4% of the total population. Malaria is hyperendemic in the region with parasite prevalence in children under 5 years of 45% during the dry season and above 70% during the rainy season [21]. Heath services are provided in the district through 6 physicians, 2 midwifes, 17 nurses, 19 matrones and 18 vaccinators.

### Study Design

The study was an open cluster-randomized trial. The 22 health areas (sub-districts) of the district of Kolokani were randomized in a 1:1 ratio with the intervention in 11 health areas and the other 11 serving as controls for the assessment of the impact of IPTi implementation on EPI vaccines and other health interventions coverage as well as its impact on the *P. faciparum *resistance to SP. IPTi was implemented from December 2006 to December 2007 and children 0-23 months were surveyed at baseline and one year after to assess of the impact of IPTi implementation on EPI vaccines and other health interventions coverage [21].

#### IPTi implementation

The intervention consisted on the administration to infants of ½ tablet of SP along with EPI vaccines (DTP2, DTP3 and Measles/Yellow fever vaccine) from December 2006 to December 2007. Prior to the randomization of the health areas, IPTi implementation tools (training modules, supports for data collection, monitoring and evaluation, information and communication) were developed by working groups including personnel of the health district, and the regional heath directorate. Supports for child health interventions (vaccination card, vaccines registration forms, monitoring and evaluation forms etc...) were modified to allow the recording of the administration of the SP along with EPI vaccines and the health interventions. Forms for procurement and supply of SP were developed to be used along with those of other health interventions. Communities' leaders were sensitized and health of the health staffs were trained.

#### Randomization

At the end of training session of staff of communities health centers, the 22 health areas (sub-districts) of the district of Kolokani were randomized into a 1:1 ratio into two zones (intervention zone and control zone). For transparency and better adhesion of the community health centers representatives, a manual randomisation was done publically as follow. The health areas were numbered from 1 to 22 and each number was written on piece of paper that was folded. The 22 pieces of paper were then mixed and placed in box and 11 of them were randomly drawn to serve as intervention areas by one of the trainees in presence of the representatives of the 22 communities' health centers (see additional file 1).

#### Assessment of the EPI vaccines and health interventions coverage

Two cross-sectional surveys were performed, one at baseline in November 2006 prior to the beginning of IPTi implementation and another in December 2007 after one year of IPTi implementation. Thirty clusters were selected using a random sampling with probability proportional to the size of the population size. In each cluster (location), a sample of about 35 children in November 2006 and about 70 children in December 2007 aged 0-23 months were randomly selected and surveyed using the WHO method of evaluation of vaccine coverage [25]. An interviewer-administered questionnaire was used to obtain data using modified UNICEF multiple indicators cluster surveys questionnaire (http://www.childinfo.org/mics2_questionnaire.html). Parents or guardians of children aged 0-23 months in selected households were questioned about the EPI vaccines and other health interventions. Information on EPI vaccination cards was recorded for each child and in its absence, questions regarding the EPI vaccines were asked and responses recorded. The information on vaccination were collected by interview only when the vaccination card was not available. When the card is not available and a parent answers a particular question as "do not know" this information is considered missing and ignored in the analysis. The interviews were conducted in local language and by two interviewers in each household. Main indicators included child immunizations and IPTi coverage, use of insecticide-impregnated bed net (ITN) during the previous night, and child receiving vitamin A supplementation. Filled questionnaires were verified at the end of each day by a supervisor and corrected if necessary.

### Endpoints

The primary endpoint was the proportion of children aged 9-23 months completely vaccinated. Children of 9-23 months were considered completely vaccinated if they had had BCG, at least 3 doses of DTP, Polio, measles and yellow fever vaccines. Secondary endpoints are: i) absolute proportions of children who received appropriate doses of each of EPI vaccines (one dose of BCG, 3 doses for Polio, 3 doses for DTP, one dose for measles vaccine, one dose of yellow fever vaccine), vitamin A supplementation, and who slept under ITN and ii) relative proportion of children who received individual doses of IPTi.

### Sample size

Sample size for the baseline survey was estimated using the following assumptions. Based on a precision of 6% and alpha error of 5% and DTP3 coverage of two thirds (67%), a sample of 472 children was selected using a cluster effect of 2. This sample size was doubled to take into account analysis for specific age categories and increased by 10% to take into account missing information, making a total sample size of 1,050 children aged 0-23 months. During the second survey after one year of implementation, the same number of 1,050 children was sampled in each zone (intervention and non intervention) to allow estimates of vaccine coverage in each zone and comparisons between them. This will provide 90% power or more based on a proportion of completely vaccinated between 50% and 67% and a non-inferiority margin of 10% with two tailed significance level of 5%.

### Data management and analysis

Data were double entered and reconciled using Epi Info version 6 (CDC Atlanta). The cleaned database was exported to Stata 9 (Houston Texas, USA) for the analysis. Vaccine coverage was determined using the information on vaccination card and from interview and was defined as the proportion of children who receive the vaccine based on the information on the card or the vaccination history provided by mothers or guardians among the total number of children surveyed in the target age group. Target age groups were defined as age ranges starting from the age at which the vaccine is given according to EPI vaccination schedule up to 23 months of age (i.e. 0-23 months for BGG and, 4-23 months for the three doses of DTP and Polio vaccines and 9-23 months for measles and yellow fever vaccines). Children of 9-23 months were considered completely vaccinated if they had had BCG, at least 3 doses of DTP, Polio, measles and yellow fever vaccines. Proportions of children who received IPTi relative to the three EPI vaccinations (DTP2, DTP3, and measles vaccines) were computed as the number of children who had documented doses 1, 2 or 3 of IPTi, divided by the number of children with documented DTP2, DTP3, and measles vaccines respectively. Coverage of IPTi1 and IPTi2 were computed for children aged 4-15 months and coverage of IPTi3 for those aged 9-15 months. Coverage of ITN was defined as the number of children who slept under ITN the night before divided by the number of children surveyed, and coverage of vitamin A supplementation was defined as the number of children 6 -23 months who received a vitamin A supplementation divided by the number of children of this age group surveyed. EPI vaccines and other health intervention's coverage were compared between the two zones at post-intervention survey and before and after the IPTi implementation. The 95% confidence intervals were adjusted for cluster design using cluster option in Stata (version 9, Houston Texas USA).

### Ethical considerations

Ethical clearance was obtained from the Ethical Committee of the Faculty of Medicine Pharmacy and Dentistry of the University of Bamako, Mali, and informed consent from a parent or legal guardian was obtained prior to the enrollment of the child in the survey.

## Results

### Participants' characteristics

At baseline, a total of 1050 children were surveyed, 51.9% were male. Vaccination card was available for 71.2% (95% CI 64.5% - 77.9%) of the children surveyed. During the post -intervention survey, a total of 1051 children were surveyed in the intervention and 1055 in the control zone. About 50.7% in the intervention zone were male and 52.8% in the control zone (P = 0.34). The proportion of children with EPI vaccination cards was similar between intervention and control areas; 78.0% (95% CI 69.4% -86.6%) versus 80.1% (75.6% - 84.6%) (P = 0.65).

### Coverage of IPTi after one year of implementation

The proportion of children in the target age groups who received IPTi with each of the 3 vaccinations DTP2, DTP3 and Measles, were 89.2% (95% CI 85.9%-92.0%), 91.0% (95% CI 87.6% -93.7%) and 77.4% (95% CI 70.7%-83.2%) respectively. The corresponding figures in non intervention zone were 2.3% (95% CI 0.9% -4.7%), 2.6% (95%CI 1.0% -5.6%) and 1.7% (95% CI 0.4% - 4.9%).

### Coverage of vaccines and other health interventions at baseline and after implementation of IPTi

Coverage of the vaccines and health intervention at baseline and after one year of IPTi implementation are presented in Table 1.

**Table 1:** Coverage of EPI vaccines given with IPTi and other health interventions based on the information on vaccination card and from interview at baseline and after IPTi implementation

Vaccines and interventions	Pre-intervention		Post-intervention				P value Control zone versus intervention zone	P value Control zone versus Baseline	P value Intervention zone versus Baseline
	Baseline		Control zone		Intervention zone				
	n	Coverage in % (95% CI)	n	Coverage in % (95% CI)	n	Coverage in % (95% CI)			
BCG	867	70.9% (62.7 - 79.1)	925	88.7% (82.6 - 94.9)	958	91.6% (88.7 - 94.6)	0.322	<0.001	<0.001
3 doses of DTP/Polio	704	54.4% (43.3 - 65.5	718	60.2% (48.6 -71.7)	763	76.4% (69.5 -83.3)	0.007	0.03	<0.001
Measles	560	55.0% (44.4 - 65.6)	544	73.9% (67.6 - 80.1)	528	83.3% (78.8 - 87.9)	0.012	<0.001	<0.001
Yellow Fever	599	49.9% (38.7 - 61.1)	544	73.2% (67.2 - 79.1)	529	82.6% (77.8 - 87.4)	0.011	<0.001	<0.001
Completely vaccinated	537	36.7% (25.3 - 48.0)	519	53.8% (43.4- 64.1)	511	69.5% (64.2 - 74.7)	0.005	<0.001	<0.001
Vitamin A supplementation	720	74.4% (67.3 - 82.5)	723	83.7% (77.1- 90.3)	691	85.2% (78.0-92.5)	0.746	<0.001	<0.001
Use of ITN	1039	49.7% (39.4 - 59.6)	1045	52.7% (41.3 - 64.1)	1045	52.3% (43.7 - 61.0)	0.884	0.17	0.24

EPI: Expanded Programme on Immunization; IPTi: Intermittent Preventive Treatment in infants; BGG: Bacillus Calmette-Guérin vaccine; DTP: Diphtheria-Tetanus-Pertussis vaccine; Polio: Poliomyelitis vaccine; ITN: Insecticide-impregnated bed net.

At baseline, based on information from interviews and vaccination cards, the proportions of children 4 -23 months who received BGG, 3 doses of DTP and Polio, measles vaccine and yellow fever vaccine were 70.9% (95% CI 62.7% -79.9%), 54.4% (95% CI.43.3% - 65.5%), 55% (95% CI 44.4% - 65.6%). The proportion of children aged 6 months and above, who received Vitamin A supplementation was 74.4% (95% CI 67.3% - 82.5%). Sleeping under ITN was reported for 49.7% (95% CI 39.4% - 59.6%) of children aged 0-23 months. The proportion of children 9-23 months who were completely vaccinated (defined as children who received BGG, 3 doses of DTP/Polio, measles and yellow fever vaccines) was 36.7% (95% CI 25.3% - 48.0%).

After one year implementation of IPTi, coverage of vaccines and vitamin A supplementation increased significantly in both control and intervention zones compared to baseline. The proportion of children completely vaccinated increased significantly from 36.7% (95% CI 25.3%-48.0%) at baseline to 53.8% (95% CI 43.4% - 64.1%) (P < 0.001) in the control zone and to 69.5% (95% CI 64.2% - 74.7%), (P < 0.001) in the intervention zone during the post-intervention survey. The use of ITN was similar in post-intervention survey compared to baseline.

With exception to BCG, the vaccines coverage during the post-intervention survey was significantly higher intervention zone compared to the non intervention zone. The proportion of children completely vaccinated was 69.5% (95% CI 64.2% -74.7%) in the intervention zone and 53.8% (95%CI 43.4% - 64.1%) in the non intervention zone (P = 0.005). The proportions of children who received Vitamin A supplementation and proportion of children using ITN were similar in the two zones.

## Discussion

This study showed a significant increase in the proportion of children completely vaccinated after one year of implementation of IPTi in the intervention zone compared to non intervention (P = 0.005) and in each of the two zones in compared to baseline (P < 0.001). The increase in vaccine coverage in the intervention zone compared to the non intervention zone occurred for all the vaccines with the exception of BCG, the vaccine that was not given with IPTi. The coverage of vitamin A supplementation and use of ITN was also similar in the two zones after one year of implementation. However, there was a significant increase in proportion of children who received vitamin A in the each of the two zones in post -intervention survey compared to baseline. Possible reasons for this higher coverage in the IPTi intervention zone compared to the non intervention zone include more adhesion of the community because of the high acceptability of the intervention, the additional motivation of the health workers and increase in supervision of EPI activities due to the introduction of IPTi-SP. Mothers' knowledge of immunization or other interventions is known to be positively correlated with full immunization rates [23,24,26] and it is possible that the adhesion of the community through information and sensitization was responsible for this increase in EPI vaccines coverage compared to baseline levels for all the vaccines and for the fact that this increase was more marked in the intervention zone compared to the non intervention zone. Acceptability surveys conducted in Mali (our unpublished data) and other countries in sub-saharan Africa [15] have found that mothers like IPTi and come more to health centers because i) they are concern with malaria; ii) they think it is an anti-pyretic (and post-vaccinal fever is one of the major bottlenecks of EPI coverage) and iii) it is free. Even in the context of maternal illiteracy, educating mothers about the vaccines and vaccine-preventable diseases was reported to be highly effective in increasing the immunization coverage [27]. Positive mother's perceptions about IPTi have been reported in previous studies [6,28].

Because in many countries in sub-Saharan Africa including Mali there is a critical shortage of health service providers [29]**, **the concern that addition of IPTi-SP to the vaccination package could result in a reduction in the coverage of the EPI vaccines and other interventions already delivered through EPI was legitimate. However, not the full time of health center's staff was used in Mali and the addition of IPTi to the current EPI package is feasible without significant uptake of EPI services as reported in Ghana [28].

As indicated in a previous study, the strategy is well accepted by the health staff. The time used for IPTi implementation is acceptable and was estimated to 12.4 minutes ranging from 1.6-28.9 minutes per nurse [30]. It represents about 11% of health workers' time [31] which is compatible with the current health staff and activities in Mali.

The relatively closer and more regular supervisions, especially at the beginning of the intervention, may have also contributed to increased staff adherence and motivation that could have resulted in an increase in EPI vaccines coverage in post-intervention compared to baseline.

In line with other reports [23,24,32] the proportion of children aged 9-23 months completely vaccinated was only 36.7% in November 2006. However this proportion increased significantly to 53.8% in non intervention and 69.5% in the intervention zone after one year of IPTi implementation. This suggest that i) low vaccination coverage should not be criteria preventing the introduction of IPTi into existent health systems; ii) IPTi can be easily added to the routine immunizations of EPI without impairing its coverage but on the contrary with the possibility of boosting it; iii) cost and effectiveness studies [4,33] and assessment of the role of IPTi-SP in infants [34-36] should consider a positive interaction between IPTi and EPI vaccine coverage.

It is possible that the staff adhesion and the enthusiasm toward this new intervention have resulted in the observed increase of EPI vaccination coverage in the control zone compared to baseline. Although there was an increase on EPI vaccine coverage in Mali 29% in 2001 to 48% in 2006 [24], the increase from 36.7% to 53.8% in the non intervention zone in one year is likely due to IPTi implementation in other parts of the district. The proportion of children completely vaccinated after the IPTi implementation in the intervention zone (69.5%) was consistent with a previous report in the district of Kita in Mali, after three years of intense EPI vaccination priority program [37].

Our study also showed that high coverage (89% or above) of IPTi can be obtained when the strategy is implemented in the health system.

To the best of our knowledge, this is the first study to assess the impact of IPTi-SP on EPI vaccine coverage using a cluster-randomized design. Additional studies undertaken as part of the UNICEF pilot implementation of IPTi-SP using pre-and post-implementation surveys, although with more potential for bias, will provide further insight on the impact of the strategy on the EPI vaccines coverage. Like any study of its kind, the possibility of founding these results by chance alone and or as results of selection bias cannot be excluded. In our study the coverage of vaccines and health intervention tended to be higher in the intervention zone at baseline although the differences were not statistically significant (see additional file 2). Nevertheless the increase in vaccines coverage in both zones compared to baseline and the fact that significantly higher coverage of vaccines given with IPTi was found in intervention zone compared to non -intervention are reassuring and indicate that the IPTi implementation has played a role. If the increase in EPI vaccines coverage is confirmed in other studies, along with the recommendations of IPTi as policy by the WHO [38], decision-makers in sub-Saharan Africa should consider rapid adoption and implementation of the strategy.

The use of ITN remained similar between the two zones and compared to baseline, suggesting that the IPTi implementation did not lower people's attitudes on malaria prevention.

Limitations of this study include i) the lack of control intervention and blinding, ii) the fact that the training was provided to staff of the all the health areas in the district including in control zone and iii) the fact that the strategy was implemented for only one year. In addition, because of the natural of the deployment, one cannot control for "implementation slips" generated by the lack of borders of community sensitization and the migration between the two zones, although the proportion of children who received IPTi in the control zone was low (< 3%). Like with any new intervention of its kind, continuous monitoring of its impact on the EPI vaccine coverage will be needed along with the implementation of the strategy over longer period of time.

## Conclusions

In summary, this study shows that high coverage of IPTi can be obtained using routine health services and IPTi-SP implementation resulted in a significant increase in coverage of EPI vaccines in the district of Kolokani, Mali, suggesting that the cost-effectiveness of the intervention may be higher than reported and that low coverage of the EPI vaccine should not preclude the implementation of IPTi-SP.

## Abbreviations

BGG: Bacillus Calmette-Guérin vaccine; DTP: Diphtheria-Tetanus-Pertussis vaccine; DTP2: Second dose of Diphtheria-Tetanus-Pertussis vaccine; DTP3: Third dose of Diphtheria-Tetanus-Pertussis vaccine; EPI: Expanded Programme on Immunization; IPTi: Intermittent Preventive Treatment in infants; ITN: Insecticide-impregnated bed net; Polio: Poliomyelitis vaccine; SP: Sulfadoxine-Pyrimethamine; UNICEF: United Nations of International Children's Emergency Fund; WHO: World Health Organization.

## Competing interests

The authors declare that they have no competing interests.

## Authors' contributions

AD was the principal investigator of the study. He contributed to the study design, oversaw the study conduct, the data collection and contributed to the data analysis and interpretation. IS, MSS, ATD, AS contributed to the study design and in overseeing the IPTi implementation. Data were collected by or under the supervision of ST and MT. CR, RS and OKD contributed to the study design and data analysis and interpretation. Data Management and quality control was performed under supervision of OBT. The manuscript was drafted by AD and all the authors contributed to revision and approved the final version.

## Pre-publication history

The pre-publication history for this paper can be accessed here:

http://www.biomedcentral.com/1471-2458/11/573/prepub

## Supplementary Material

Additional file 1 **Map of the health areas of the district of Kolokani, Mali**. The figure is a map of district of Kolokani, showing the health areas (sub districts) with the intervention areas are indicated in red. Click here for file

Additional file 2 **Coverage of EPI vaccines by zone at baseline**. The table summarizes the coverage of EPI vaccines and other health interventions based on the information on vaccination card and from interview at baseline in intervention and control zones. Click here for file
