# Supplementary material for: Increase in EPI vaccines coverage after implementation of intermittent preventive treatment of malaria in infant with Sulfadoxine -pyrimethamine in the district of Kolokani, Mali: Results from a cluster randomized control trial
Source: BMC Public Health. 2011 Jul 18;11:573. doi: 10.1186/1471-2458-11-573 (PMC3155918; doi:10.1186/1471-2458-11-573)

Additional file 1. Map of the health areas (sub districts) of the district of Kolokani, Mali. Intervention areas are indicated in red.

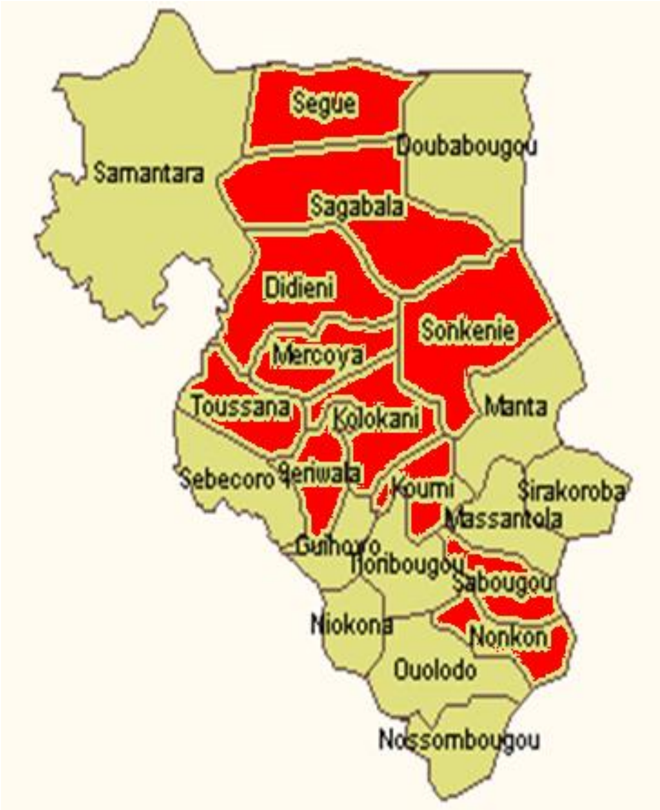

Supplement: Additional file 1 — Map of the health areas of the district of Kolokani, Mali. The figure is a map of district of Kolokani, showing the health areas (sub districts) with the intervention areas are indicated in red. [file 1471-2458-11-573-S1.PDF]
