# Supplementary material for: Increase in EPI vaccines coverage after implementation of intermittent preventive treatment of malaria in infant with Sulfadoxine -pyrimethamine in the district of Kolokani, Mali: Results from a cluster randomized control trial
Source: BMC Public Health. 2011 Jul 18;11:573. doi: 10.1186/1471-2458-11-573 (PMC3155918; doi:10.1186/1471-2458-11-573)
Supplement: Additional file 2 — Coverage of EPI vaccines by zone at baseline. The table summarizes the coverage of EPI vaccines and other health interventions based on the information on vaccination card and from interview at baseline in intervention and control zones. [file 1471-2458-11-573-S2.PDF]

Additional file 2: Coverage of EPI vaccines and other health interventions based on the information on vaccination card and from interview at baseline in intervention and control zones.

Vaccines	No IPTi		IPTi		p
	n	Coverage in % (95% CI)	n	Coverage in % (95% CI)	
BCG	410	67.3 (53.7 - 80.9)	457	74.2 (65.0 - 83.4)	0.39
3 doses of DTP	332	55.7 (38.3 - 73.1)	377	66.6 (54.3 - 78.8)	0.34
3 doses de Polio	340	51.8 (42.0 - 74.3)	375	59.2 (55.4 - 79.6)	0.50
3 doses of DTP/Polio	331	51.7 (33.7 - 69.6)	373	56.8 (43.5 - 70.2)	0.47
Measles	263	51.0 (35.1 - 66.9)	297	58.6 (44.9 - 72.3)	0.46
Yellow Fever	263	46.8 (29.1 - 64.4)	296	52.7 (39.9 - 66.5)	0.59
Completely vaccinated	249	33.3 (16.2 - 50.4)	288	39.6 (24.8 - 54.3)	0.58
Vitamin A supplementation	347	69.2 (57.5 - 80.7)	373	79.4 (70.2 - 88.6)	0.17
Use of ITN	523	44.2 (27.5 - 60.8)	516	55.2 (45.3 - 65.2)	0.26
